# Deoxyhypusine Hydroxylase from *Plasmodium vivax*, the Neglected Human Malaria Parasite: Molecular Cloning, Expression and Specific Inhibition by the 5-LOX Inhibitor Zileuton

**DOI:** 10.1371/journal.pone.0058318

**Published:** 2013-03-07

**Authors:** Veronika Anyigoh Atemnkeng, Mario Pink, Simone Schmitz-Spanke, Xian-Jun Wu, Liang-Liang Dong, Kai-Hong Zhao, Caroline May, Stefan Laufer, Barbara Langer, Annette Kaiser

**Affiliations:** 1 Institute of Pharmacogenetics, University of Duisburg-Essen, Essen, Germany; 2 Occupational Medicine, University of Duisburg-Essen, Essen, Germany; 3 State Key Laboratory of Agricultural Microbiology, Huazhong Agricultural University, Wuhan, PR China; 4 Immune Proteomics, Medizinisches Proteom-Center, Ruhr-University Bochum, Bochum, Germany; 5 Pharmazeutische Chemie, Pharmazeutisches Institut, Eberhard-Karls-Universität Tübingen, Tübingen, Germany; Centro de Pesquisa Rene Rachou/Fundação Oswaldo Cruz (Fiocruz-Minas), Brazil

## Abstract

Primaquine, an 8-aminoquinoline, is the only drug which cures the dormant hypnozoites of persistent liver stages from *P. vivax*. Increasing resistance needs the discovery of alternative pathways as drug targets to develop novel drug entities. Deoxyhypusine hydroxylase (DOHH) completes hypusine biosynthesis in eukaryotic initiation factor (eIF-5A) which is the only cellular protein known to contain the unusual amino acid hypusine. Modified EIF-5A is important for proliferation of the malaria parasite. Here, we present the first successful cloning and expression of DOHH from *P. vivax* causing tertiary malaria. The nucleic acid sequence of 1041 bp encodes an open reading frame of 346 amino acids. Histidine tagged expression of *P. vivax* DOHH detected a protein of 39.01 kDa in *E. coli.* The DOHH protein from *P. vivax* shares significant amino acid identity to the simian orthologues from *P. knowlesi* and *P. yoelii* strain H. In contrast to *P. falciparum* only four E-Z-type HEAT-like repeats are present in *P. vivax* DOHH with different homology to phycocyanin lyase subunits from cyanobacteria and in proteins participating in energy metabolism of *Archaea* and *Halobacteria*. However, phycocyanin lyase activity is absent in *P. vivax* DOHH. The *dohh* gene is present as a single copy gene and transcribed throughout the whole erythrocytic cycle. Specific inhibition of recombinant *P. vivax* DOHH is possible by complexing the ferrous iron with zileuton, an inhibitor of mammalian 5-lipoxygenase (5-LOX). Ferrous iron in the active site of 5-LOX is coordinated by three conserved histidines and the carboxylate of isoleucine^673^. Zileuton inhibited the *P. vivax* DOHH protein with an IC_50_ of 12,5 nmol determined by a relative quantification by GC/MS. By contrast, the human orthologue is only less affected with an IC_50_ of 90 nmol suggesting a selective iron-complexing strategy for the parasitic enzyme.

## Introduction

The human malaria parasite *Plasmodium vivax* is responsible for 25–40% of the 515 million annual cases of malaria worldwide. It is the major cause of human malaria outside of Africa and occurs mainly in Asia and the Americas [1]. Although this infection is seldom fatal, the parasite elicits severe clinical symptoms and often causes relapses after a primary infection has cleared [2]. These relapses are initiated by blood-stage infections due to persisting dormant hypnozoites for months or even years in hepatocytes of the liver. Hypnozoites survive most drugs that kill blood-stage parasites. Hitherto, primaquine [3] is the only licensed drug which affects the hypnozoites and radically cures from the infection. However, resistance to the drug is spreading [4]. Moreover, its use is contra-indicated in pregnant women and patients with glucose-6-phosphate dehydrogenase deficiency, which is common in malaria-endemic regions.

Despite its importance as a major human pathogen, knowledge about *P. vivax* is rather limited because it cannot be propagated continuously *in vitro* except in non-human primates. Several biological characteristics underlie the distinct pathogenic nature of *vivax* malaria. In contrast to *P. falciparum*, *P. vivax* is only capable of infecting reticulocytes, causing severe anemia by dyserythropoiesis and destruction of infected and uninfected erythrocytes despite much lower parasitemias. Although recent data from the *P. vivax* genome consortium [5] shed more light on the biology of this neglected human malaria parasite i.e. a highly conserved metabolome, and 77% identity of gene orthologues, the knowledge about the dormant hypnozoites remains rather inadequate. Studies of the hypnozoite transcriptome would give a significant contribution in understanding the signals which switch the dormant form of the hypnozoite into its infectious form. However, this investigation remains technically challenging since the needed biological material is missing at present. Currently efforts are underway to establish new *in vitro* culture systems in human hepatocytes [6]. These distinct biological features have delayed research to find drugs against *P. vivax*.

For over 50 years the treatment of *Plasmodium vivax* has relied on a combination of chloroquine plus primaquine. However, both drugs are under threat because of increasing resistance. By contrast, artemisinin combination therapy (ACT) is only effective against the erythrocytic stages of drug resistant *P. vivax*, while it does not protect against relapses. Primaquine is the only drug which radically cures hypnozoites but requires long treatment [7].

**Figure 1:**
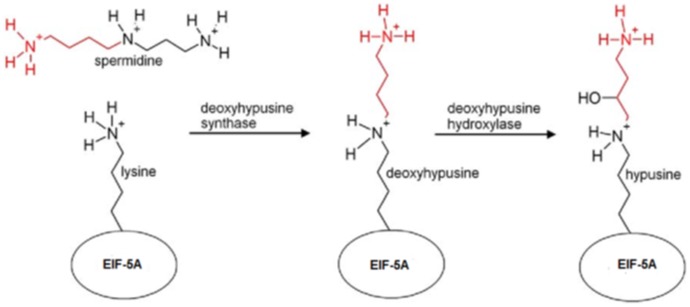
Hypusine is formed within two subsequent steps. The first step is catalysed by deoxyhypusine synthase which transfers an aminobutyl moiety from the triamine spermidine to eukaryotic initiation factor 5A in a NAD^+−^dependent reaction. Hypusine biosynthesis is completed by the hydroxylation of the side chain by deoxyhypusine hydroxylase.

During the last years we have investigated the biosynthetic pathway (Fig. 1) of the unusual amino acid hypusine in eukaryotic initiation factor (eIF-5A) in different malaria parasites for drug discovery of antimalarials. EIF-5A is a ubiquitous, cellular protein in eukaryotes being involved in translation elongation [8], [9] rather than in translation initiation. Recent results identified the *eIF-5A* gene as a tumour suppressor gene [10]. Hypusine is formed by the catalysis of two different enzymatic steps. In the first step the transfer of an aminobutyl moiety from the triamine spermidine to a specific lysine in eIF-5A is catalysed by deoxyhypusine synthase (DHS) while deoxyhypusine hydroxylase (DOHH) completes hypusine biosynthesis by hydroxylation. Hitherto, in *Apicomplexa* the *dohh* gene has only been recently identified and functionally expressed from *P. falciparum* strain NF54 [11]. DOHH from *P. falciparum* contains several significant matches to E-Z HEAT-like repeats present in E/F-type phycocyanin lyases of cyanobacteria and red algae [12]. Phycobiliproteins, unlike other light-harvesting proteins are involved in photosynthesis, bear covalently attached chromophores. The bilin chromophores are attached through thioether bonds to cysteine residues of the apoproteins by phycocyanin lyases which then organize into phycobilisomes the light harvesting supercomplexes of cyanobacteria and red algae.

Although the amino acid identity of *P. vivax* DOHH to the human orthologue is rather low i.e. 27%, the histidine-glutamate (HE) motifs at the active site of the enzyme which coordinate metal-chelating sites are highly conserved. In the past these findings were used for an iron-chelating strategy of the human enzyme [13]. Purified human recombinant DOHH is a mixture of active holoenzyme containing 2 mol of iron/mol of DOHH and inactive metal-free apoenzyme. The metal chelation of Fe^2+^ in human DOOH was performed by a panel of metal chelators including mimosine, 2,2′-dipirydyl, deferiprone, deferoxamine, and ciclopiroxolamine showing significant inhibition of human DOHH [14], [15]. However, the iron complexing strategy failed in case of the *P. falciparum* protein [15]. For antimalarial therapy an iron-chelating inhibitor with a significant lower K_i_ for the plasmodial enzyme would be of interest.

Zileuton, (RS)-1-(1-Benzothiophen-2-ylethyl)-1-hydroxyurea, [16] is a selective inhibitor of 5-lipoxy-genase (5-LOX) from human which converts arachidonic acid (AA) into leukotriens. LOX metabolites are potent physiological effectors in a variety of cellular responses associated with normal host defense and inflammation. In the 5-LOX pathway leukotriens (LTs) are the mediators of allergy and asthma. After activation of leukocytes arachidonic acid is released from the membrane by the action of cytosolic phospholipase A_2_ which in turn binds 5-lipoxygenase-activating protein (FLAP). An increase of calcium concentration leads to translocation of 5-LOX into the membrane where it finally acquires its substrate arachidonic acid.

Polyunsaturated fatty acids are the substrates of lipoxygenases. Mammalian 5-LOX performs oxygenation at position C5 in arachidonic-acid where it inserts molecular oxygen stereospecifically at the ‘S’ position [16].

Zileuton [17] chelates the active, iron containing site of the 5-lipoxygenase. All LOX have a two-domain structure, the small N-terminal ß-barrel domain and a larger catalytic domain (LOX domain) containing a single atom of non-heme iron. The catalytic iron is ligated in an octahedral arrangement by three conserved histidines (amino acid positions 367,372 and 550 in stable human 5-lipoxygenase) and the main chain carboxylate C-terminal isoleucine (amino acid position 673) [18]. Based on the data obtained from a structural human 5-lipoxygenase model, DOHH from *Plasmodium* shows similarities to human 5-lipoxygenase in its active site where four conserved histidine glutamate residues (amino acid residues 75–76, 108–109, 250–251, 282–283) coordinate the ferrous iron. DOHH is a non-heme diiron enzyme. Therefore, the drug was applied to investigate specific inhibition of plasmodial DOHH.

Hitherto, we have not understood the role of hypusine in the infection process of the benign and severe malaria parasite. Since the DOHH protein from *P. falciparum* has been assessed as a druggable target in *P. falciparum* and *Leishmania major* [19] we now describe the molecular cloning of the *dohh* orthologue from the neglected human malaria parasite *P. vivax* for target evaluation. Purified DOHH protein shows functional activity and has only four E-Z HEAT-like repeats opposed to five compared to its orthologue from *P. falciparum.* Moreover, we demonstrate that the active catalytic site of DOHH from *P. vivax* can be selectively inhibited by zileuton, an inhibitor of 5-lipoxygenase. This specific effect might reflect a common mechanism resulting from iron complexation of the inhibitor in both enzymes.

## Materials and Methods

### Amplification of the *dohh* Gene from Genomic DNA of *P. vivax* Salvador PEST-1 Strain

The PCR reaction for amplification contained in a volume of 20 µl 200 pmol genomic DNA from *P. vivax* Salvador PEST-1 strain, 200 pmol of each primer *P. vivax dohh* primer # forward 5′-ATG ACA GGA AGT ACT CAC-3′ and *P. vivax dohh* primer reverse # 5′-TCA ATT TAC TTC TAT TGC C-3′, dNTP (10 mM), MgCl_2_ (75 mM), 2 µl PCR-buffer (10 fold), 4 µl Q-Solution (5-fold) (Qiagen) and 5 U Taq polymerase (Qiagen). Amplification was performed using a PCR with a temperature profile of 94°C of 5′, 30 cycles at 94°C of 1′, 50°C of 2′, 72°C of 2′, and a final elongation step at 72°C. The amplified PCR product of 1041 bp was directly cloned into pSTBlue-1 AccepTor vector (Novagen) and positive clones were verified by dideoxynucleotide sequencing (Eurofins MWG, Munich).

### Subcloning of the *P. vivax dohh* Gene into the Histidine Tagged pET-28 Expression Vector

The recombinant *dohh* clone was used as a template for a subsequent amplification step with restriction sites for *Not*I # dohh expression primer forward # 5′- GCG GCC GCA TGA CAG GAA GTA CTC-3′ and *Bam*HI # dohh expression primer reverse # 5′ - ATT TGG ATC CTC AAT TTA CTT C- 3′, respectively. The amplified PCR fragment was ligated into *Not*I and *Bam*HI digested pET-28a respectively and the construct was resequenced. The restriction sites are underlined.

### Northern Blot Analysis of *P.vivax dohh*

Blood from an infected patient with *P. vivax* was applied for a short–term culture [20] and synchronized [21]. Total cellular parasitic RNA from the different developmental stages was isolated with the Plant RNeasy Kit (Qiagen, Hildesheim, Germany). Northern blots were performed with the digoxigenin-labeled full-length cDNA and quantified densitometrically (Biostep, Wolferstadt Germany).

### Expression of the *P. vivax* DOHH Protein and Purification by Nickel-chelate Chromatography

*E.coli* BL21(DE3) cells containing the recombinant *dohh* plasmid vector pET-28a (Novagen, Merck 4 Biosciences, Darmstadt Germany) were grown for expression in kanamycin (15 µg^.^mL). One millilitre samples from the expressing strain were taken and centrifuged at 13 000 rpm for 2 min. Cells were lysed with 400 µl lysis buffer (50 mM Tris/HCl, pH 8.0 2 mM EDTA), centrifuged, resuspended in lysis buffer and sonicated twice at 4°C for 30 s (tip 1 at 50% using a Branson sonifier). After centrifugation for 10 min at 16 000 rpm at 4°C, samples were diluted 1∶1 in loading buffer (20 mM Tris, pH 6,8, 2% (w/v) SDS, 2 mM EDTA, 20% (V/V) glycerol, 0,3% bromphenol blue) heated at 100°C and run on a 10% SDS polyacrylamide gel at 100 V.

Protein purification was performed by nickel-chelate affinity chromatography under native conditions, according to the manufacturer’s (Qiagen, Hilden Germany) protocol with some variations. A pellet from a 25 ml culture of *dohh* expressing *E. coli* BL21 (DE3) cells was resuspended in 630 µl lysis buffer containing pH 8.0. Lysozyme stock solution (70 µl of 10 mg.mL^−1^) and 3 U.mL^−1^ culture volume Benzoanase® Nuclease were added. The suspension was incubated on ice for 15–30 minutes. Centrifugation was applied at 12,000×g for 30 min at 4°C. A Ni-NTA spin column was equilibrated with 600 µl lysis buffer containing 10 mM imidazole. Centrifugation for 2 min at 890×g followed. Six hundred microliters of the cleared lysate containing the 6x His-tagged protein was loaded onto the pre-equilibrated Ni-NTA spin column and centrifuged for 5 min at 270×g. The Ni-NTA spin column was washed twice with 600 µl washing buffer containing 5.0 mM NaH_2_PO_4_, 300 mM NaCl, 5 mM imidazole, pH 8.0 and centrifuged for 2 min at 890×g. DOHH was eluted from the column with 300 µl elution buffer containing 50 mM NaH_2_PO_4_, 300 mM NaCl, 500 mM imidazole, pH 8.0 in two fractions.

### Detection of Proteins by Silver Staining

Detection of proteins was performed with the Silver-staining kit according to a protocol from Roth (Karlsruhe, Germany). After gel electrophoresis, protein gels were fixed, sensitized and silver impregnated before they were developed.

### Non-radioactive Detection of the Hypusine Metabolites by Gas Chromatography Mass Spectrometry

Since hypusine is formed by two subsequent enzymatic reactions i.e. deoxyhypusine synthase and deoxyhypusine hydroxylase, the first reaction step i.e. the formation of EIF-5A ^(Dhp)^ was performed with the N-terminal histidine tagged fusion proteins of EIF-5A and DHS in recombinant pET-15b expressed in *E.coli* BL21(DE3) cells. Purification was performed by nickel-chelate chromatography with subsequent buffer exchange with a Sephadex-G25 column for the activity assay. The reaction mixture of 1 mL contained spermidine, EIF-5A from *P. vivax* (40 µM), 0.5 mM NAD^+^, and 2.6 µg purified DHS enzyme and was incubated at 30°C for 2 h. Enrichment of deoxyhypusinylated eIF-5A (Dhp) was performed within two steps of size-exclusion chromatography with a Microcon YM-100 cutting off DHS and a subsequent step with Microcon YM-30. EIF-5A was analysed by subsequent peptide hydrolysis for deoxyhypusine modification. The typical DOHH assay contained in a reaction volume of 600 µl: 20 µg eIF5A (Dhp), purified DOHH enzyme from *P. vivax* (7,5 µg), 50 mM sodium phosphate buffer pH 7.4, 1 mM NAD, 1 mM DTT. Incubation was performed for 3 h at 37°C. Subsequently, the completely modified hypusinylated EIF-5A protein was recovered by size-exclusion chromatography as previously described to cut off DOHH. Samples were hydrolyzed for 24 h at 110°C in 10 M HCl. As a control hydrolyzed BSA was used.

### Radioactive DOHH Activity Assay

A typical assay for DOHH activity contained 20 mM Tris-HCl pH 7.5, 6 mM DTT, 1 mg/ml BSA, EIF-5A [^14^-C-Dhp] [22], [23], and 14 µl H_2_O in a volume of 20 µl. The volume of the enzyme was 20 µl. The reaction was incubated for 2 h at 37°C. The reaction was stopped by adding 5 µl (250 µg) of carrier BSA and 0.2 ml of ice cold TCA solution, mixed and kept on ice for 20 min. The sample was centrifuged in a refrigerated microfuge at 4°C at 15.000×g for 5 min. The supernatant was removed and radioactivity was counted. The pellet was treated with 0.4 ml 6 M HCl and heated in a heating block at 108°C overnight and analysed further by GC/MS analysis.

### GC/MS Analyses

GC/MS analysis was performed using an HP 6890 gas chromatograph and a 5973 quadrupole mass spectrometer (Agilent Technologies, Santa Clara, CA, USA). The instrument was equipped with a 30 m×320 µm (i.d.) Optima 1 column coated with a 10% dimethylpolysiloxane cross-linked stationary phase (0.25 µm film thickness; MACHEREY-NAGEL, Düren, Germany). Helium was used as carrier gas (flow rate of 1.5 mL/min). The analytes (2 µL each) were injected in the splitless mode with a solvent cutoff time of 6 min. The injector temperature was maintained at 250°C. The oven temperature was kept at 60°C for 1 min and then linearly increased at a rate of 5°C/min up to 280 where it was maintained for 5 min. The MS was operated in the electron impact (EI) ionization mode at 70 eV with the quadrupole temperature set at 150°C and the source temperature at 230°C. Full scans were acquired by repetitively scanning over the mass range from 50 to 550 Da at a scan rate of 500 msec/scan. Identification of deoxyhypusine was performed after acidic hydrolysis and derivatization with methyl chloroformate, according to the prominent fragment peaks 87 m/z, 129 m/z and 143 m/z. Hypusine was identified according to the prominent fragments 88 m/z, 101 m/z and 157 m/z.

### Determination of Phycocyanin Lyase Activity

Genes *cpcA, pecA* from *Anabaena* PCC7120 were cloned into pETDuet, *ho1 plus pcyA* from *Anabaena* PCC7120 were cloned into pACYCDuet, and *cpcE, cpcF, pecE, pecF*, *cpcE* plus *cpcF*, *pecE* plus *pecF* from *Anabaena* PCC7120 were cloned into pCDFDuet, respectively [24]. The *dohh* gene was a recombinant construct in pET28a as described previously (see Material and Methods within). For expression dual plasmids were transformed together into *E. coli* BL21(DE3) which were grown in 100 ml LB medium with the appropriate antibiotic at 37°C until an OD600 of 0.6 was reached. When grown to an OD600 of 0.5–0.6, the cultures were incubated for 30 min at 4°C, and then IPTG was added to a final concentration of 1 mM. After expression at 20°C for 12 hrs, cells were harvested.

The cell pellets were resuspended in ice-cold potassium phosphate buffer (KPB, 20 mM, pH 7.0) containing 0.5 M NaCl, and disrupted by sonication for 5 min at 200 W (JY92-II, Scientz Biotechnology, Ningbo, China). The suspension was centrifuged at 12,000× *g* for 15 min at 4°C, and the supernatant purified *via* Ni^2+^-affinity chromatography on chelating Sepharose (Amersham Biosciences), developed with KPB containing 0.5 M NaCl. Bound proteins were eluted with the above saline KPB containing, in addition, imidazole (0.5 M). After collection, the sample was dialyzed twice against the saline KPB. The relative lyase activity of was assayed by the amount of the assembled products, i.e. chromophorylated CpcA or PecA, which is investigated with UV-Vis absorption spectroscopy (Beckman-Coulter DU 800).

## Results

### Molecular Cloning and Characterization of the *dohh* Gene from *P. vivax*

Based on the identified open reading frame of the nucleic acid sequence from *P. falciparum dohh* a bio-informatics screening of the *P. vivax* genome [5] was performed.

From the nucleic acid sequence obtained, we constructed two gene-specific primers for the 5′ and 3′ prime end and amplified a 1041 bp fragment encoding an ORF of 346 amino acids on chromosome 14. The putative *dohh* gene from *P. vivax* is an AT-rich gene with a content of 65% in comparison to the GC-content of 34.6%. The nucleic acid sequence of the *dohh* gene from *P. vivax* is closely related to its orthologues from the simian and human parasite *P. knowlesi* (92% identity) [25] and the rodent malaria parasite *P. yoelii* strain H (78% identity). The nucleic acid sequence of *P. vivax* is deposited in the EMBL database under the accession number AM931168.

**Figure 2:**
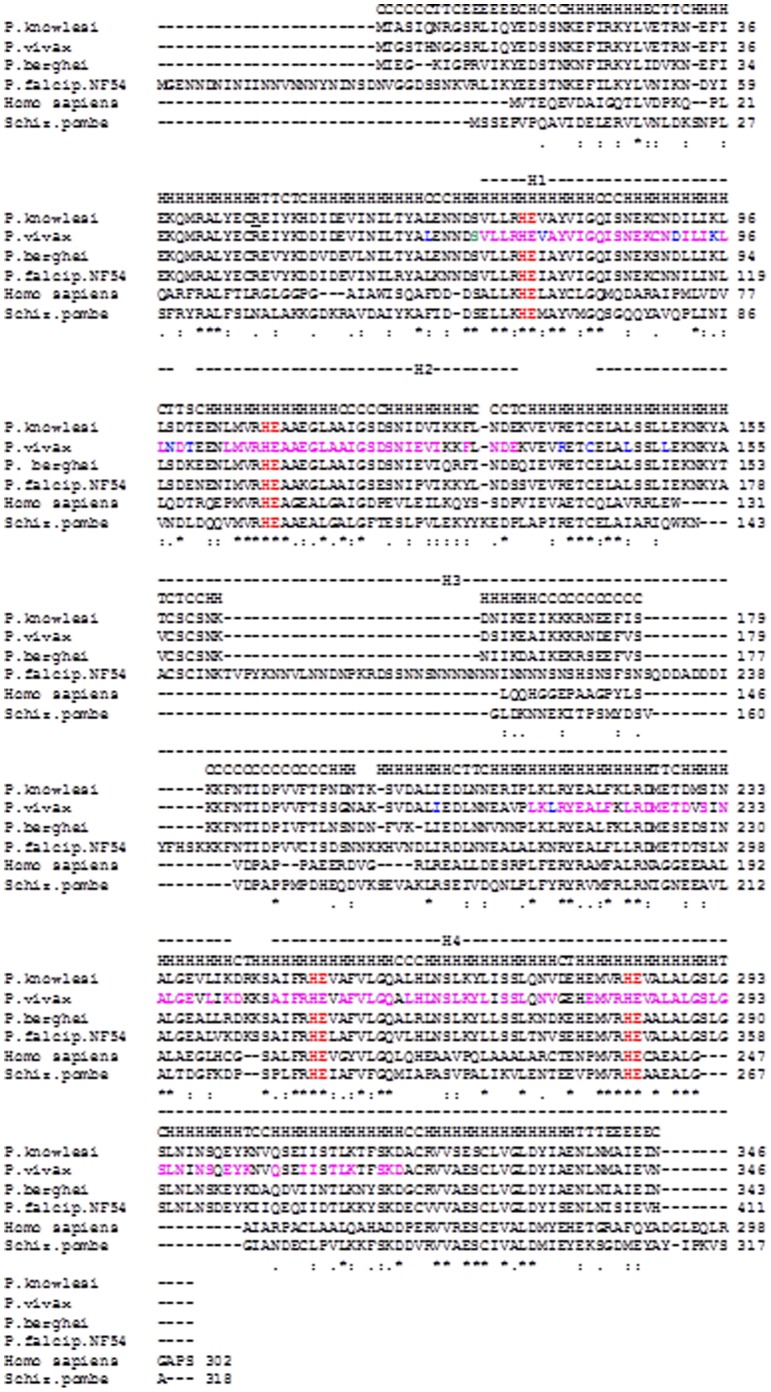
A phylogram of *P. vivax* DOHH summarizes the evolutionary relationship of the protein in different *Plasmodium* species. PHYLIP and CLustalW programmes were applied for the protein alignment and calculation of the tree distances. Tree distances are given in numbers in brackets. The most common recent ancestors of *P. vivax* DOHH protein are orthologues from the simian/human malaria parasite *P. knowlesi* and the rodent malaria parasite *P. berghei*. The evolutionary tree interrelationship of DOHH from *P. vivax* to its human and *P. falciparum* orthologue is low.

**Figure 3:**
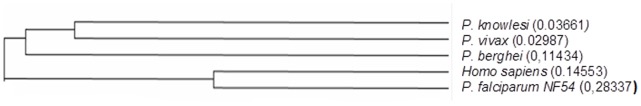
Multiple amino acid alignment of DOHH from *P. vivax* to three different *Plasmodium* species (*Plasmodium knowlesi*, *Plasmodium berghei* and *Plasmodium falciparum*). Human DOHH and DOHH from *P. vivax* were also included in the alignment. EZ-HEAT repeats are numbered in their sequential order and are coloured in pink. Significant is the amino acid identity of blue coloured amino acids in EZ-HEAT repeat number 1 (amino acid position 68–96) and EZ-HEAT repeat number 2 (amino acid position 103–134) to EF-type phycocyanin lyase from cyanobacteria [38].The other two different EZ-HEAT repeats exhibit various degrees of identity to enzymes involved in energy metabolism (significant amino acids are marked in blue letters). The highly conserved histidine glutamate residues are marked in red. The secondary structure prediction is given above the alignment. The secondary structure is presented on top of the alignment and was determined by the JPred vers. 3.0 and Scratch programmes [39]. H represents the α-helix, E means an extended structure, T means a β-turn, and C symbolizes the remainder. Gaps (.) were introduced to obtain maximal alignment. Asterisks label amino acid identities, colons (:) and dots (.) label amino acid similarities.

The amino acid sequence of *P. vivax* DOHH was aligned to amino acid sequences from different *Plasmodium* species. Significant amino acid identities were discovered for *P. knowlesi,* and *P. berghei* (95%) while there is less amino acid identity in case of *P. falciparum* (67%) (Fig. 2). The yeast and human orthologues were also included in the alignment and showed 44% and 32% amino acid identity respectively. DOHH from *P. vivax* has a molecular weight of 39.01 kDa and an isoelectric point of 4.88. The different plasmodial DOHH proteins show common structural features (Fig. 2) i.e. the occurrence of E-Z-type HEAT-like repeat motifs. However, in contrast to the DOHH protein from *P. falciparum* [10] we identified only four E-Z-type HEAT-like repeat motifs located at amino acid positions 58–146 (H1), 71–100 (H2), 104–133 (H3) and 232–294 (H4) in DOHH from *P. vivax* (Fig. 3). All four E-Z-type HEAT-like repeats have significant homology to HEAT-like repeat domains SM00567 (SMART), pfam 13646 present in phycocyanin lyases from cyanobacteria. In contrast to DOHH from *P. falciparum*, a screen based on conserved domains in *P. vivax* DOHH identified COG1413, a HEAT-repeat like domain involved in energy production and conversion which occurs in proteins of the *Archaea Methanosarcina acetovirans* (Methanosarcinaceae), *Halobacterium* Sp. NRC-1 (Halo-bacteriaceae) and in PBS lyase HEAT-like repeat proteins from different enterotoxic *E. coli* strains (http://www.ncbi.nlm.nih.gov/Structure/). Both *P. vivax* and *P. falciparum* DOHH contain a highly conserved histidine-glutamate (HE) motif coordinating the ferrous iron in the centre of the active site.

A phylogram which was constructed from different DOHH amino acid sequences of various species using the CLUSTAL W and PHYLIP programs (Fig. 3) revealed insights into the phylogenetic relationship between *P. vivax* DOHH and its orthologues from various organisms. DOHH from *P. vivax* (tree distance: 0,02987) has the closest relationship to DOHH from the rodent and human parasite *P. knowlesi* with a calculated tree distance of 0.03661. However, the relationship between the DOHH protein from *P. vivax* (tree distance: 0,02987) and *P. falciparum* (tree distance: 0,28337) is significantly lower suggesting that its common ancestor is from the rodent and simian parasites.

### Transcription of the *Plasmodium vivax dohh* Gene in different Developmental Stages of the Infection Process

**Figure 4:**
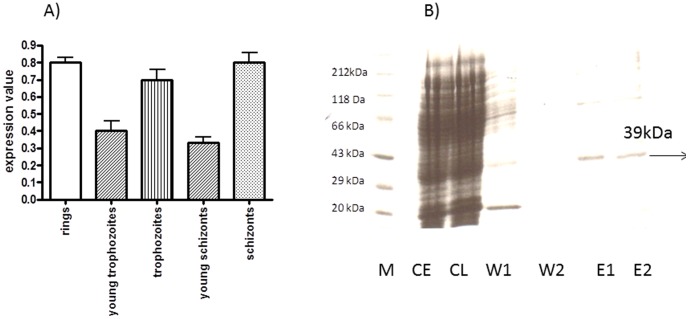
A: Transcriptional profile of *P. vivax dohh* throughout the 48 h intraerythrocytic life cycle. Cellular RNA was isolated from a clinical isolate obtained from a patient infected with *P. vivax* at different developmental stages of the infection during the intraerythrocytic life cycle. Expression values were determined for all given developmental stages (x-axis) in comparison to an average value obtained from a housekeeping gene (y-axis). B: Purification of *P. vivax* DOHH by Nickel-chelate-affinity chromatography. Lane 1) Standard protein marker from Roth (Karlsruhe, Germany); Lane 2) Bacterial lysate; Lane 3) Crude extract; Lane 4) Wash fraction 1; Lane 5) Wash fraction 2; Lane 6) Eluate fraction 1; Lane 7) Eluate fraction 2. The SDS-PAGE gel was stained by Coumassie Blue.

Next, we investigated the transcriptional profile of the *P. vivax dohh* gene throughout the 48 h intraerythrocytic life cycle performing Northern Blot analyses with cellular RNA isolated from a clinical isolate. Transcription of the *dohh* gene could be constantly observed throughout the whole intraerythrocytic cycle of 48 h (Fig. 4A). Transcription values of approximately 0.8% were detected in the trophozoite stage (Fig. 4A) while transcription decreased to 0.4% within the early schizont stage. A similar pattern of transcription was detected in late developmental stages i.e. in schizonts between 30 to 48 hours where transcript formation decreased to 0.3% in young schizonts before it increased to 0.8% in mature schizonts. These results are in agreement with a transcriptional profile of a database entry published in PlasmoDB [http://plasmodb.org/plasmo/] of a hypothetical protein Pvx_121970 from three patients where the putative *dohh* gene is even transcribed in sporozoites. However, our investigations were focused on the erythrocytic stages.

### Expression, Purification and Functional Analysis of *P. vivax* Deoxyhypusine Hydroxylase

The histidine-tagged *dohh* construct was expressed in pET-28a under the control of the T7 promotor in *E. coli* BL21(DE)3 cells harbouring the T7-RNA polymerase. DOHH from *P. vivax* was expressed as a protein with a molecular size of 39.1 kDa (Fig. 4B). Maximum levels of DOHH expression were detected after 3 hours of induction. Purification of the histidine tagged DOOH protein was performed by Ni-chelate affinity chromatography. Under native conditions the protein eluted in both eluate fractions (Fig. 3B, lanes E1 and E2).

**Figure 5:**
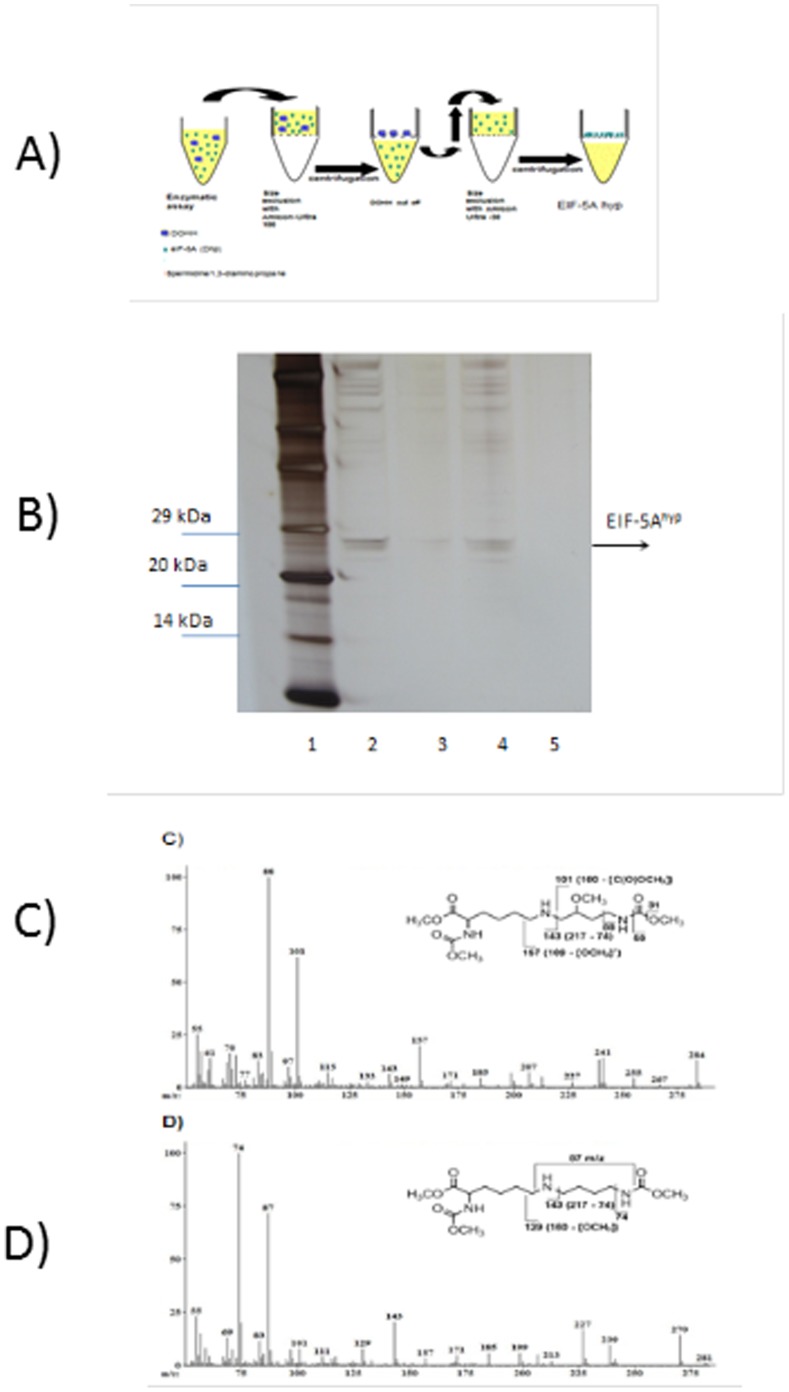
A: Schematic overview of the DOHH activity assay by two steps of size exclusion chromatography. B: Silver staining of a protein gel after size exclusion chromatography of DOHH activity assays with Amicon-Ultra 100 and Amicon-Ultra 30 columns. Modified EIF-5A protein was recovered in the eluates after size exclusion chromatography with Amicon Ultra 30 columns. Lane 1) Standard protein marker from Roth (Karlsruhe, Germany); Lane 2) Recovered, modified EIF-5A protein after the DOHH activity assay with the inhibitor zileuton (100 nmol); Lane 3) Flowthrough of Amicon Ultra 30 column after the DOHH activity assay with the inhibitor zileuton (100 nmol); Lane 4) Recovered, modified EIF-5A protein of a non-inhibitor treated DOHH activity assay; Lane 5) Flowthrough of a non-inhibitor treated DOHH activity assay; Identification of (C) hypusine and (D) deoxyhypusine by GC/MS analysis after DOHH activity assays obtained from a peptide hydrolysate of modified EIF-5A protein after derivatization with methyl chloroformate. Hypusine was identified by the prominent molecular fragments 88 m/z, 101 m/z and 157 m/z in the ion mass spectrum. Deoxyhypusine was identified by the prominent molecular fragments 87 m/z, 129 m/z and 143 m/z (Fig.5D). Break points of the molecule are indicated by vertical lines while horizontal lines indicate the fragment and its corresponding mass, e.g. the deoxyhypusine fragment 87 m/z represents a [NH(C_4_H_8_)NH]^+^ fragment and the 74 m/z fragment the terminal [NHC(OO)CH_3_] group.

Next we monitored hydroxylase activity in a non radioactive assay. Since the specific activity of human deoxyhypusine synthase is significantly higher than the parasitic enzyme the human, orthologous protein was applied in the first step of eIF-5A modification. Nickel-chelate-affinity chromatography was used to purify human DHS which is able to modify the parasitic EIF-5A precursor protein. The deoxyhypusinylated, modified EIF-5A precursor protein was enriched in two subsequent steps of size exclusion chromatography using an Amicon-Ultra 100 and an Amicon-Ultra 30 column (Fig. 5A) as described previously [10]. DHS was cut off after the first step of size exclusion chromatography. Column fractions were analysed by SDS-PAGE and checked by silver staining (Fig. 5B). The modified EIF-5A^dhp^ occurred in the Amicon-Ultra 100 and Amicon-Ultra 30 eluates (Fig. 5A). The eluate from the Amicon-30 column contained the enriched deoxyhypusinated EIF-5A^dhp^. EIF-5A^dhp^ was applied as a substrate for the DOHH activity assay. Again DOHH was cut off by two steps of size exclusion chromatography and hypusinated EIF-5A^hyp^ was analysed further after peptide hydrolysis and derivatization with methyl chloroformate by GC/MS (Fig. 5C). Methyl chloroformate derivatizes the reactive side chains and esterifies the carboxylic groups. The authentic deoxyhypusine standard showed three prominent peaks of 87 m/z, 129 m/z and 143 m/z after hydrolysis. Hypusine was identified by its prominent peaks 88 m/z, 101 m/z and 157 m/z after hydrolysis.

In a separate experiment we performed the DOHH activity assay with 100 nmol of the 5-lipoxgenase inhibitor zileuton and identified the presence of modified EIF-5A^hyp^ and its precursor EIF-5A^dhp^ by GC/MS analysis. The activity assay revealed a deoxyhypusine to hypusine ratio of 90.4% to 9.6% based on the peak areas while the determined deoxyhypusine to hypusine ratio in the non-treated DOHH was 47% to 53%, respectively.

**Figure 6:**
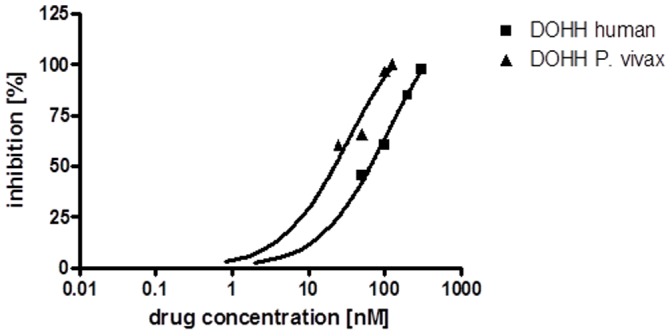
Dose response curves of zileuton obtained after inhibition of DOHH from *P. vivax* and *P. falciparum.* Different zileuton concentratons from 5 to 300 nmol were applied. The percentage of inhibiton was plotted against the different drug concentrations. The dose-response curve of *P. vivax* DOHH is presented in triangles and that for human DOHH in squares.

To compare the inhibitory effect of zileuton on human and parasitic DOHH inhibition the radioactive filter assay was applied since GC/MS analysis only allows a relative quantification. Zileuton applied in a concentration of 100 nmol inhibited parasitic DOHH approximately 9 fold while only 1.3 fold inhibition was detected for the human enzyme (Table 1). These results were furthermore substantiated by a dose-response curve with various zileuton concentrations inhibiting either recombinant DOHH from *P. vivax* or the human orthologue. Surprisingly, zileuton was more effective in inhibition of the parasitic enzyme in comparison to the human orthologue (Fig. 6). Moreover, zileuton resulted in a determined IC_50_ value of 90 nmol for the human DOHH protein while the IC_50_ value of 12,5 nmol was significantly lower for the *P. vivax* protein (Table 2).

**Table 1:** Determination of DOHH activity from *P. vivax* and human.

A	B	C	D	E	F
**EIF-5A^Dhp^**	Water	EIF-5A^Dhp^	EIF-5A^Dhp^	EIF-5A^Dhp^	water
**14-[C]-spermidine**	14-[C]-spermidine	14-[C]-spermidine	14-[C]-spermidine	14-[C]-spermidine	14-[C]-spermidine
**P.vivax DOOH**	P.vivax DOOH	P.vivax DOOH	Human DOOH	Human DOHH	Human DOHH
		Zileuton (100 nmol)			Zileuton (100 nmol)
**1050 U/mg DOHH protein ±10 U**	–	105 U/mg DOHH protein ±5 U	1900 U/mg DOHH protein ±18 U	1520 U/mg DOHH protein ±20 U	–

Column A) Complete DOOH activity assay with *Plasmodium* DOHH; B) Control Assay: DOHH protein from *P. vivax* was substituted by water; C) Inhibitor assay: DOHH from *P. vivax* was inhibited with 100 nmol zileuton; Column D) Complete DOOH activity assay with human DOHH; Column E) Control Assay: DOHH protein from human was substituted by water; F) Inhibitor assay: DOHH from human was inhibited with 100 nmol zileuton. Each experiment was performed in three replicates the same day.

**Table 2:** IC_50_ values of different iron chelators determined in *in vitro* cultures of *P. falciparum* and human cell lines compared to IC_50_ values after zileuton inhibition of recombinant human and *P. vivax* DOHH.

Drug	Mimosine	Mimosine		ciclopirox	ciclopirox	4-Oxo-piperidine-3-mono- carboxylate	Zileuton	zileuton
**Culture/protein**	*P.faciparum in vitro culture*		HUVEC*	*P. falciparum in vitro culture*	HUVEC*	*P. falciparum in vitro culture*	DOHH *P.vivax*	DOHH *P.vivax*
**IC_50_ [µM]**	32–39 µM [40]		191 µM [40]	8,2 µM [40]	5 µM [40]	1,7 µM [40]	12,5 nmol [40]	90 nmol [40]

Data from mimosine, ciclopirox, 4-Oxo-piperidine-3-mono- carboxylate were taken from [40].

* HUVEC = Human umbicilial Vein Endothelial Cells.

### Determination of Phycocyanin Lyase Activity

**Figure 7:**
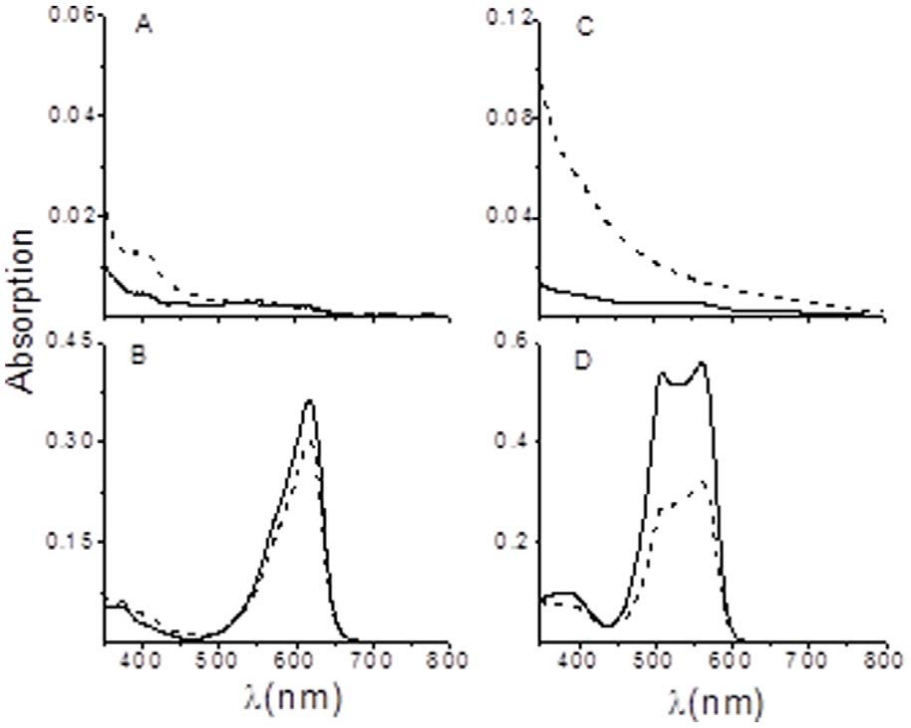
Assay of DOHH for phycocyanin α-84 lyase and phycoerythrocyanin α-84 lyase / isomerase activities. Absorption spectra of acceptor proteins, CpcA and PecA, after treatment with PCB in the presence (–) or absence (–) of DOHH, and purification by Ni^2+^ affinity chromatography. (A) Assay for the attachment of PCB to CpcA. (B) In the additional presence of the lyase, CpcE /F. (C) Assay for PCB isomerizing to PVB and attachment to PecA. (D) In the additional presence of the isomerizing lyase, PecE /F. All reactions were carried out in *E. coli* (see Materials and methods for details).

Since DOHH from *P. vivax* shares two EZ-HEAT-like repeats present in phycocyanin lyase from various species we tested the expressed DOHH protein for a possible, dual E/F type phycobilin lyase activity (Fig. 7). Recombinant plasmids expressing the acceptor proteins CpcA or PecA and the PCB catalyzing enzymes [heme oxygenase (HO1) and PCB:ferredoxin oxidoreductase (PcyA) were co-transformed together with expression plasmids encoding the non-isomerising lyase, CpcE/F or the isomerising lyase, PecE/F and the recombinant *dohh* plasmid respectively, into *E. coli* strain BL21 (DE3). Heme oxygenase (HO1) oxygenates heme to biliverdin while PcyA reduces biliverdin to phycocyanobilin (PCB). Thus both reactions lead to PCB. Controls were performed in the absence or presence of expressed DOHH protein. Addition of the apoprotein to the chromophore was determined by absorption spectroscopy. The chromophorylated CpcA or PecA were purified by Sepharose chelating chromatography. In Fig. 7A the control experiment of the residual, spontaneous addition of the CpcA to ring A of the chromophore was tested, which was minimal on assembly in *E. coli*. The spontaneous (non enzymatic) chromophore addition generates an absorption at 645 nm [26]. The isomerized product of the phycoerythrocyanin lyase, phycoviolobilin, is characterized by an absorption at ∼565 nm for the Z-form and 505 nm for the E-form (Figure 7D). Figures 7A and C show no attachment of the chromophore to the apoprotein irrespective of the presence or absence of DOHH. In the presence of the phycocyanin α-84 lyase the chromophore was properly attached (Fig. 7B) while the presence of recombinant plasmodial DOHH had no effect. Similar results were obtained when an additional isomerizing lyase, PecE/F encoding phycoerythrocyanin α-84 lyase/isomerase was present (Fig. 7D). Here, the DOHH protein had a slightly inhibitory effect in comparison to the control (Fig. 7C) which shows the attachment of isomerized phycoviolobilin to the apoprotein.

**Figure 8:**
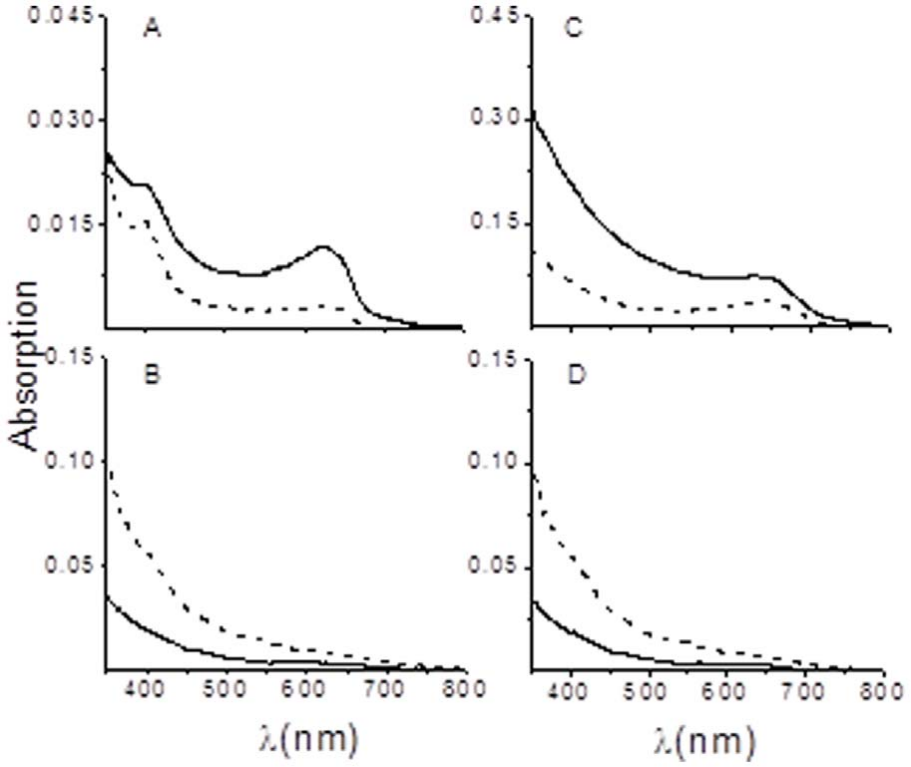
Assay of DOHH for phycocyanin α-84 lyase and phycoerythrocyanin α-84 lyase activities. Absorption spectra of acceptor proteins, CpcA and PecA, after treatment with PCB in the presence (–) or absence (–) of DOHH, and purification by Ni^2+^ affinity chromatography. (A) Assay for the attachment of PCB to CpcA in the additional presence of the lyase, CpcE. (B) Assay for the attachment of PCB to CpcA in the additional presence of the lyase, CpcF; (C) Assay for the attachment of PCB to PecA in the additional presence of the lyase, PecE. (D) Assay for PCB isomerizing to PVB and attachment to PecA in the additional presence of the lyase, PecF. All reactions were carried out in *E. coli* (see Materials and methods for details).

In the next set of experiments the different subunits of the non-isomerizing lyase CpcE or CpcF, or the different subunits of the isomerizing lyase PecE or PecF in the presence or absence of the recombinant DOHH protein were tested for a possible activity of DOHH which might be similar to the subunit of E/F type phycobiliprotein lyase (Fig. 8). Fig. 8A shows the absorption spectrum for the attachment of the chromophore PCB to the apoprotein CpcA in the presence of the additional lyase subunit CpcE. In comparison to the absorption spectra obtained when both subunits of the non-isomerizing lyase CpcE/F are present (Fig. 8B) absorption is significantly lower when only one subunit either CpcE or CpcF is present (Fig. 8A,B). In case of the additional CpcE or CpcF subunit, absorption spectra were not influenced in the presence or absence of plasmodial DOHH. Moreover, no lyase activity could be detected when either PecE (Fig. 8C) or PecF (Fig. 8D) were present. Based on these experiments we conclude that DOHH from *P. vivax* has no phycocyanin lyase activity.

### Approaches for an in Silico Homology Modelling of *P. vivax* DOHH

In an attempt to design a homology model for *P. vivax* DOHH we performed a screening for a suitable template in different databases. Screening with the Protein Model Portal resulted in one hit to the 3HGC protein an acid- sensing eukaryotic ion channel protein which is proton-activated [27]. The aligned protein is an amiloride-sensitive cation channel transport protein from *Gallus gallus* with a sequence identity of 31% on the amino acid level. However, homology modelling resulted in a model of poor quality.

A parallel screen performed with Swiss model resulted in the template of YIBA a predicted HEAT-repeat containing lyase from *E. coli* [28] with an amino acid sequence identity of 19% as previously described [12]. Again, the constructed model did not show the expected quality.

Next the amino acid sequence of stable human 5-lipoxygenase was aligned to *P. vivax* DOHH. Both proteins i.e. human 5-LOX and DOHH from *P. vivax* are rich in α-helices [Fig. 2]. The overall amino acid identity is 21% between both proteins. Again,3 D-modeling failed because of less quality between both proteins due to the low amino acid identity.

## Discussion and Conclusions

Here, we report on the cloning of the deoxyhypusine hydroxylase gene from the neglected, benign human malaria parasite *P. vivax.* DOHH from the benign malaria parasite encodes an ORF of 346 amino acids and thus differs in its length and in the number of structural motifs from its *P. falciparum* orthologue [11]. DOHH from *P. vivax* has a close relationship to the rodent parasites *P. knowlesi* (97%) and *P. yoelii* (78%) and no significant relationships to the orthologues from parasites belonging to the kinetoplastids i.e. trypanosomes and leishmania.

In contrast to the *P. falciparum dohh* gene with five E-Z-HEAT like repeats, *P vivax* has only four E-Z- HEAT- like repeat domains. However, in both *Plasmodium* species two E-Z HEAT-like repeat domains recruit from phycocyanin lyases present in different cyanobacteria [29], [30]. During evolution *Plasmodium* acquired the apicoplast which is a relic of the chloroplast from cyanobacteria [31]. The *dohh* gene is not located in the apicoplast genome but retained structural elements which show its recruitment from phycocyanin lyase which is an enzyme involved in photosynthesis from cyanobacteria. Therefore, it might be tempting to speculate that during evolution these two phycocyanin lyase derived E-Z HEAT-repeats in DOHH provide an extensive soluble surface that is well suited to interact with a different tetrapyrrolic chromophore rather than phycocyanobilin. An attractive candidate could be hemoglobin being a precursor of biliverdin chromophores. Although DOHH has lost its phycocyanin lyase activity it might be a potential binding protein of hemoglobin which is essential for the parasite to maintain its amino acid requirement [32]. Experiments to investigate binding of DOHH to hemoglobin are currently under way [33].

The two other HEAT-domains which occur in *P. vivax* DOHH derive from a COG1413 domain present in *Methanosarcina* from *Archae and Nostoc* from *Cyanobacteria*. The basis of the evolutionary success of these HEAT-like repeats might be the rapid adaptation to different interacting partners [30]. At the sequence level this is reflected in the extent of sequence divergence that can be observed for the individual repeats. In consequence, beside EIF-5A as the main interacting partner for DOHH from *P. vivax*, different interacting partners might be involved depending on the cellular responses and environmental stimuli. In this context it is interesting to note that a recent tandem affinity based protein complementation study demonstrated a significant interaction [34] between human DOHH and LDH-A (lactate dehydrogenase isoenzyme A) (P06151) and pyruvate kinase isoenzymes M1/M2 (P52480). Lactate dehydrogenase catalyzes the interconversion of either lactate or pyruvate with concomitant interconversion of NADH and NAD^+^. Under facultative anaerobic or anaerobic conditions lactate dehydrogenase converts lactate to pyruvate and the reverse reaction is catalyzed during the Cori cycle. In both reactions NAD^+^ is regenerated from NADH which is used for continuation of glycolysis. In sum, the HEAT-repeat domains identified in DOHH might provide the accessible surface for an interaction with both enzymes i.e LDH-A and pyruvate kinase to maintain energy metabolism.

The occurrence of two E-Z HEAT-like repeat domains present in phycocyanin lyases from different cyanobacterial species prompted us to test the DOHH protein from *P. vivax* for residual phycocyanin lyase activity. Within two different sets of experiments in the additional presence of the nonisomerizing lyase CpcE/CpcF or the isomerizing lyase, pecE/F, the recombinant DOHH exhibited no phycocyanin lyase activity (Fig. 7, 8).

Given that DOHH exerts its catalytic mechanism as a subunit of lyase activity, the additional presence of the isomerizing subunits CpcE or F and PecE or PecF respectively, had no effect (Fig. 8). Thus it seems likely that during evolution when photosynthesis was not necessary anymore parasitic DOHH has lost this function.

DOHH from *P. vivax* was expressed under the control of the IPTG-inducible T7 promotor in *E. coli* BL21 (DE3) cells. The overexpressed protein had a molecular size of 39 kDa (Fig. 3B, E1, E2) and an isoelectric point of 5.13.

*P. vivax* DOHH protein displayed deoxyhypusine hydroxylase activity in a novel, non radioactive assay [12] (Fig. 5) which was analyzed further either by GC/MS (Fig. 5) or one dimensional protein gelelectrophoresis and subsequent mass spectrometry (data not shown). It was demonstrated that the drug zileuton which is an inhibitor of human 5-lipoxygenase (5-LOX) inhibited parasitic DOHH significantly (Table 1). These experiments were even more supported by the dose-response curve (Fig. 6) comparing the inhibitory effect of different zileuton concentrations against human DOHH and *P.vivax* DOHH. The fact that DOHH from *P. vivax* is inhibited more selectively than the human orthologue might result from a higher affinity of the inhibitor to the active site of the parasitic enzyme. This is even more strengthened by the determined IC_50_ values for DOHH from *P. vivax* i.e. 12,5 nmol and for its human orthologue i.e. 90 nmol, respectively (Table 2). Moreover, the E-Z-HEAT- like repeat domains might contribute to an improved binding.

Human 5-lipoxygenase catalyzes the first two reactions in the production of leukotriens from arachidonic acid. Moreover, 5-LOX is a validated target for antiinflammation drug design. Many different inhibitors of 5-LOX have been reported like redox, iron ligands and nonredox inhibitors but only few maintain the *in vivo* activity so far. The only 5-LOX inhibitor on the market is zileuton which is used for the treatment of asthma [35]. In human whole blood, zileuton inhibited 5-LOX at a concentration of 3.3+0.4 µM while in *vivo* the weak potency and the rapid clearance are the therapeutic drawbacks.

All lipoxygenases are homologous in sequence and have the same two domain structure which is an N-terminal ß-barrel domain and a C-terminal catalytic domain (lipoxygenase domain). A catalytic iron atom resides in the C-terminal domain. Moreover, the catalytic iron is ligated in an octahedral arrangement by three conserved histidines, one His/Asn/Ser, and the C-terminal isoleucine. By contrast the ferrous iron in *Plasmodium* DOHH is coordinated by four histidine glutamate residues. The structural similarities between 5-LOX and plasmodial DOHH might explain the selective iron complexing strategy. There are recent reports [36] about a comparative modelling of the human 5-LOX inhibitor binding structure which was used to perform a virtual screen to discover novel 5-LOX inhibitors. This strategy is pursued for the parasitic enzyme although currently a crystal structure is missing. This virtual screening will combine molecular docking and pharmacophore mapping to define structure relationships.

Another interesting observation derives from a comparison of different iron-chelating compounds (Table 2) [40] tested against the *P. falciparum* DOOH. These data show that zileuton inhibits DOHH from *P. vivax* in a nanomolar range while all the other compounds unfold inhibition at a micromolar concentration. These results further support the notion of a specific inhibition by zileuton rather than the other compounds, i.e. mimosine, cyclopirox or 4-Oxo-piperidine-3-mono- carboxylate. *In vivo* experiments in the future will delineate whether the selectivity can be confirmed.

A major advantage of zileuton is its specific inhibition of the parasitic enzyme rather than the human orthologue. In an *in vitro* assay zileuton inhibited the parasitic enzyme to 90% while inhibition of the human enzyme was 10% (Table 1). Obviously, zileuton [N-(1-benzo[b]thien-2-ylethyl)-N-hydroxyurea] is a highly selective inhibitor of plasmodial DOHH. Since no crystallized structure of the human or parasitic enzyme exists the reaction mechanism of the inhibitor has to be elucidated. Another advantage of the drug is the anti-inflammatory property which will be tested for the treatment of cerebral malaria in the near future where host-specific immune and anti-inflammatory mechanisms may be important in response to the presence of parasites in the CNS [37].
